# Improved Safety and Anti-Glioblastoma Efficacy of CAT3-Encapsulated SMEDDS through Metabolism Modification

**DOI:** 10.3390/molecules26020484

**Published:** 2021-01-18

**Authors:** Hongliang Wang, Lin Li, Jun Ye, Wujun Dong, Xing Zhang, You Xu, Jinping Hu, Rubing Wang, Xuejun Xia, Yanfang Yang, Dujia Jin, Renyun Wang, Zhihui Song, Lili Gao, Yuling Liu

**Affiliations:** 1 State Key Laboratory of Bioactive Substance and Function of Natural Medicines, Institute of Materia Medica, Chinese Academy of Medical Sciences & Peking Union Medical College, Beijing 100050, China; wanghl@imm.ac.cn (H.W.); llin@imm.ac.cn (L.L.); yelinghao@imm.ac.cn (J.Y.); dwujun@vip.sina.com (W.D.); zhangxingYWS@163.com (X.Z.); youxupumc@gmail.com (Y.X.); hujp@imm.ac.cn (J.H.); wangrubing@imm.ac.cn (R.W.); xjxia@imm.ac.cn (X.X.); yangyf@imm.ac.cn (Y.Y.); djjin@imm.ac.cn (D.J.); wry@imm.ac.cn (R.W.); 2 Beijing Key Laboratory of Drug Delivery Technology and Novel Formulation, Institute of Materia Medica, Chinese Academy of Medical Sciences & Peking Union Medical College, Beijing 100050, China; zhhsong2009@126.com (Z.S.); gaolili@imm.ac.cn (L.G.)

**Keywords:** phenanthroindolizidine, self-microemulsifying drug delivery system, metabolism modification, lymphatic transport

## Abstract

13*a*-(*S*)-3-pivaloyloxyl-6,7-dimethoxyphenanthro(9,10-*b*)-indolizidine (CAT3) is a novel oral anti-glioma pro-drug with a potent anti-tumor effect against temozolomide-resistant glioma. 13*a*(*S*)-3-hydroxyl-6,7-dimethoxyphenanthro(9,10-*b*)-indolizidine (PF403) is the active in vivo lipase degradation metabolite of CAT3. Both CAT3 and PF403 can penetrate the blood–brain barrier to cause an anti-glioma effect. However, PF403, which is produced in the gastrointestinal tract and plasma, causes significant gastrointestinal side effects, limiting the clinical application of CAT3. The objective of this paper was to propose a metabolism modification for CAT3 using a self-microemulsifying drug delivery system (SMEDDS), in order to reduce the generation of PF403 in the gastrointestinal tract and plasma, as well as increase the bioavailability of CAT3 in vivo and the amount of anti-tumor substances in the brain. Thus, a CAT3-loaded self-microemulsifying drug delivery system (CAT3-SMEDDS) was prepared, and its physicochemical characterization was systematically carried out. Next, the pharmacokinetic parameters of CAT3 and its metabolite in the rats’ plasma and brain were measured. Furthermore, the in vivo anti-glioma effects and safety of CAT3-SMEDDS were evaluated. Finally, Caco-2 cell uptake, MDCK monolayer cellular transfer, and the intestinal lymphatic transport mechanisms of SMEDDS were investigated in vitro and in vivo. Results show that CAT3-SMEDDS was able to form nanoemulsion droplets in artificial gastrointestinal fluid within 1 min, displaying an ideal particle size (15–30 nm), positive charge (5–9 mV), and controlled release behavior. CAT3-SMEDDS increased the membrane permeability of CAT3 by 3.9-fold and promoted intestinal lymphatic transport. Hence, the bioavailability of CAT3 was increased 79% and the level of its metabolite, PF403, was decreased to 49%. Moreover, the concentrations of CAT3 and PF403 were increased 2–6-fold and 1.3–7.2-fold, respectively, in the brain. Therefore, the anti-glioma effect in the orthotopic models was improved with CAT3-SMEDDS compared with CAT3 in 21 days. Additionally, CAT3-SMEDDS reduced the gastrointestinal side effects of CAT3, such as severe diarrhea, necrosis, and edema, and observed less inflammatory cell infiltration in the gastrointestinal tract, compared with the bare CAT3. Our work reveals that, through the metabolism modification effect, SMEDDS can improve the bioavailability of CAT3 and reduce the generation of PF403 in the gastrointestinal tract and plasma. Therefore, it has the potential to increase the anti-glioma effect and reduce the gastrointestinal side effects of CAT3 simultaneously.

## 1. Introduction

Glioblastoma multiforme (GBM), the most common and lethal malignant central nervous system (CNS) tumor, has a poor clinical outcome [1]. Currently, temozolomide (TMZ) is the most commonly used alkylating drug as a first-line agent for GBM [2]. However, its associated toxicity and ensuing drug resistance limits the efficacy and clinical use. Therefore, it is necessary to develop more novel therapeutic drugs.

**Figure 1:**
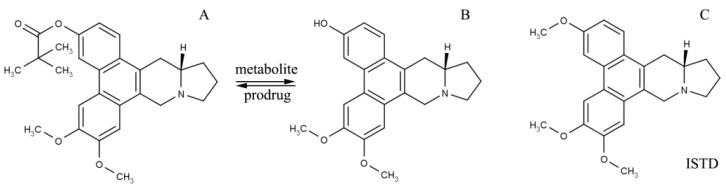
Molecular structure of CAT3 (**A**) as the pro-drug, PF403 (**B**) as the active in vivo metabolite, and (+)-Deoxytylophorinine (CAT, (**C**)) as the internal standard of the drug concentration test in plasma.

13*a*-(*S*)-3-pivaloyloxyl-6,7-dimethoxyphenanthro(9,10-*b*)-indolizidine (CAT3, Figure 1A) is a novel hedgehog signaling pathway inhibitor synthetized by Professor Yu and Professor Chen (Institute of Materia Medica, Chinese Academy of Medical Sciences & Peking Union Medical College) [3,4]. It has a significant inhibitory effect on both GBM- and TMZ-resistant GBM in orthotopic glioblastoma mice models when administrated orally [3]. Moreover, CNS toxicity, one of the main obstacles to the development of phenanthroindolizidines, has not been associated with CAT3 [5]. Previous studies reported that CAT3 is metabolized by lipase to its active metabolite, 13*a*(*S*)-3-hydroxyl-6,7-dimethoxyphenanthro (9,10-*b*)-indolizidine (PF403, Figure 1B), in vivo [3,6,7]. Additionally, the active metabolite, PF403, can penetrate the blood–brain barrier after the oral administration of CAT3 and exert a significant anti-glioma effect in vivo [8].

However, CAT3 is a biopharmaceutics classification system IV drug, which is insoluble in water and has low bioavailability in vivo [8,9]. Moreover, it demonstrates serious gastrointestinal side effects after oral administration, including severe loose stool or diarrhea, which limits its clinical application. These side effects may be ascribed to excess amounts of PF403 in the gastrointestinal tract and plasma. Furthermore, it has been reported that CAT3 is not stable in intestinal fluid and about 59% of CAT3 is metabolized into PF403 after incubation with artificial intestinal fluid for 15 min [8]. Previous research has shown that oleic acid–CAT3 conjugate-loaded solid lipid nanoparticles (OA-CAT3-SLN) can greatly increase its bioavailability [9]; however, more serious gastrointestinal side effects were observed in vivo. Therefore, simply relying on increasing bioavailability to promote therapeutic effects will not work. Furthermore, it is necessary to apply a suitable drug delivery system (DDS) for the encapsulation of CAT3 to reduce oral gastrointestinal side effects and improve the anti-glioma effect. At present, some studies have proposed methods of using a DDS, such as nanoparticles [10,11], to increase the therapeutic effect while reducing toxicity. However, this is inconsistent with the results of the CAT3 solid lipid nanoparticles mentioned above. A study using self-microemulsion to deliver cisplatin [12] attracted our attention. This DDS increased the anti-tumor effect while achieving the effect of reducing toxicity, which caused our thinking.

A self-microemulsifying drug delivery system (SMEDDS) is a lipid-based formulation DDS. It is an isotropic mixture of oil, emulsifiers, co-emulsifiers, and the drug substance. SMEDDS can increase the oral bioavailability of compounds [13,14,15], regulate hydrophobicity surfaces [16,17], and promote the uptake and transport of compounds [18,19]. SMEDDS can rapidly form nanoscale emulsion droplets spontaneously in an aqueous medium [20], such as water or gastrointestinal fluid, and encapsulate the active pharmaceutical ingredient (API) present in the medium [13]. Such encapsulation behavior can prevent API direct contact with the gastrointestinal mucosa and reduce mucosal irritation [21]. In addition, SMEDDS is mainly absorbed through the small intestine by lymphatic transport [18,22,23]; it avoids the hepatic first-pass metabolism [21] and reduces the generation of metabolites in plasma. Therefore, SMEDDS may be a suitable DDS for CAT3 to improve absorption and reduce oral gastrointestinal side effects that rely on the metabolism modification of CAT3. It will reduce PF403 in the gastrointestinal fluid or plasma and improve the accumulation of CAT3 and PF403 in the brain tissue simultaneously.

In this study, CAT3-SMEDDS (CAT3-loaded self-microemulsifying drug delivery system) was prepared and assessed for physicochemical properties and in vitro cellular uptake. Furthermore, the pharmacokinetic characteristics of CAT3 and its main active metabolite, PF403, in plasma and brain tissue were studied in vivo. The preliminary safety, anti-glioma effect, and possible mechanisms were also investigated.

## 2. Results

### 2.1. Dispersibility Test, Droplet Size, PDI, and Zeta Potential

The dispersibility test showed that CAT3-SMEDDS, after diluting with distilled water 10–1000 times, formed a fine bluish-white nanoemulsion in less than 1 min.

**Figure 2:**
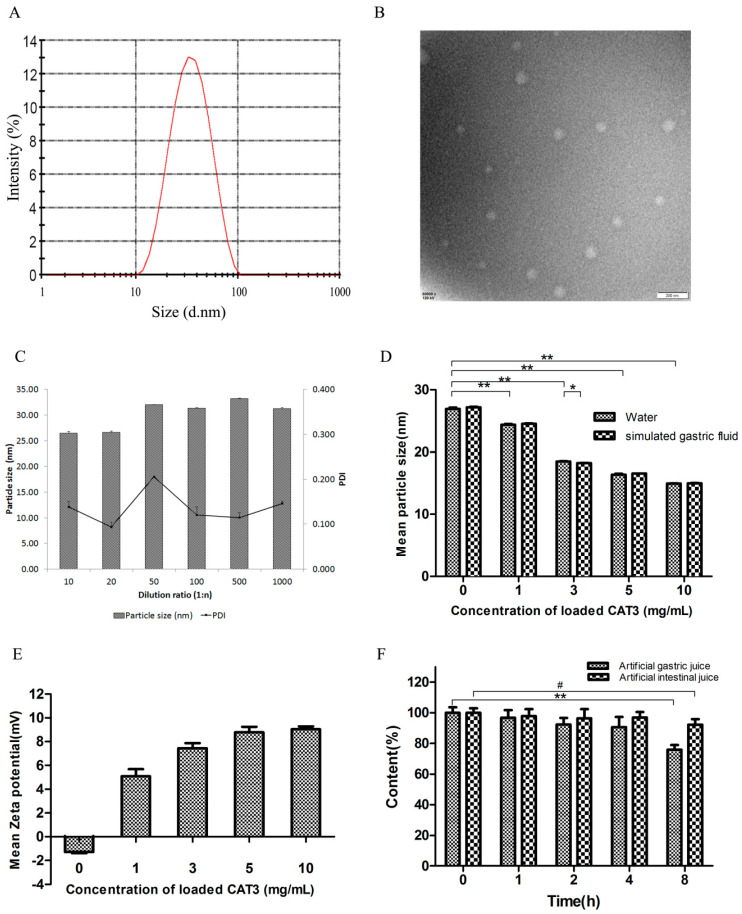
Characterization of the CAT3-loaded self-microemulsifying drug delivery system (CAT3-SMEDDS). (**A**) The typical size distribution of CAT3-SMEDDS after being diluted with water 1000-fold at 37 °C; (**B**) the TEM image of CAT3-SMEDDS nanoemulsions; (**C**) effects of different ratios of water dilution on the droplet size of CAT3-SMEDDS (*n* = 3); (**D**) effects of drug loading on the droplet size of CAT3-SMEDDS in different media (* *p* < 0.05, ** *p* < 0.01; *n* = 3); (**E**) the zeta potentials of different concentrations of CAT3 loaded in SMEDDS (*n* = 3); (**F**) the content of CAT3-SMEDDS in the artificial gastric/intestinal juice at different times (37 °C; ** *p* < 0.01, ^#^ *p* < 0.05; *n* = 3). Abbreviations: PDI, polydispersity index; CAT3-SMEDDS, CAT3-loaded self-microemulsifying drug delivery system.

Droplet size is a critical factor for evaluating a self-microemulsion system, and it affects the release and absorption profile in the gastrointestinal tract. The smaller the droplet size, the larger the interfacial surface area provided for drug absorption [24]. The typical size distribution is shown in Figure 2A. The results also show that the dilution volume had little effect on droplet size within the investigated range (Figure 2C). In addition, there was no significant difference between the distilled water and simulated gastric fluid (SGF) dilution media with regard to the droplet size (Figure 2D; *p* > 0.05). However, the mean droplet size decreased with increasing concentration of CAT3: the mean droplet size of CAT3-SMEDDS at 1 mg/mL was 26.93 ± 0.22 nm which decreased to 14.94 ± 0.05 nm at 10 mg/mL CAT3-SMEDDS (Figure 2D; *p* < 0.05). CAT3 molecule may act as an interfacial active substance to reduce the droplet particle size. The polydispersity index (PDI) of each formula and dilution ratio was less than 0.3.

Figure 2E shows the results of the droplet zeta potential of 0–10 mg/g CAT3-SMEDDS. A negative charge was displayed on the blank SMEDDS, whereas there was a positive charge on CAT3-SMEDDS. The higher the concentration of CAT3 loaded, the higher the positive charge. The changed charge value, from negative to positive, may be attributable to the tertiary nitrogen structure in the CAT3 molecular at neutral pH.

A transmission electron microscopy image is shown in Figure 2B. CAT3-SMEDDS became emulsion droplets when diluted with distilled water (1:100, *w*/*w*), and the droplets were spherical in shape.

### 2.2. The Stability of CAT3-SMEDDS in Artificial Gastrointestinal Fluid

The stability of CAT3-SMEDDS in SGF and simulated intestinal fluid (SIF) is shown in Figure 2F. In both solvents, CAT3 was stable with more than 90% drug recovery in 4 h. Moreover, CAT3 was less degradable in SIF than in SGF.

### 2.3. CAT3 Release Study

**Figure 3:**
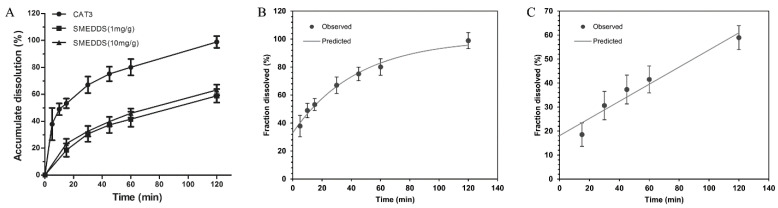
Release curves of CAT3-SMEDDS. (**A**) Release curves of CAT3 and CAT3-SMEDDS at 37 °C; (**B**) first order with the T_lag_ model of CAT3 release; (**C**) zero order with the T_lag_ model of CAT3-SMEDDS release. Abbreviations: CAT3, CAT3 suspension; SMEDDS, CAT3-loaded self-microemulsifying drug delivery system.

Figure 3 shows the cumulative drug release from the dissolution media. Unlike CAT3 (80% released in the first 60 min), CAT3-SMEDDS exhibited a sustained release profile of CAT3. More specifically, only 40% of the loaded CAT3 was released from the SMEDDS formulation in the first 60 min, and almost 60% CAT3 was released from the SMEDDS within 2 h. This sustained release profile significantly increased the absorption of CAT3, maintained a higher level of CAT3 in the blood, and facilitated a prolonged retention time. In addition, it is worth noting that there was no significant difference in the release trends between 1 and 10 mg/g CAT3-SMEDDS. The release profile of the CAT3 suspension and CAT3-SMEDDS conformed to the first order with the T_lag_ model equation (Formula (1) and Figure 3B, r = 0.9927) and zero order with the T_lag_ model equation (Formula (2) and Figure 3C, r = 0.9739), respectively.

(1) $$F=100\times \left(1-e^{-0.023\times \left(t+17.621\right)}\right),$$

(2) F=0.357×(t−(−51.414)),

### 2.4. Transepithelial Transport of CAT3-SMEDDS

**Figure 4:**
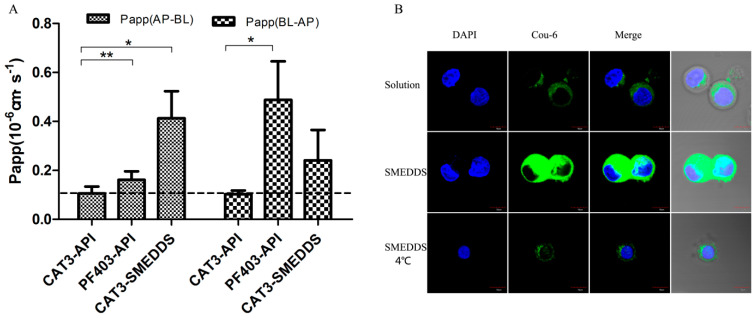
Monolayer permeability and cellular uptake of SMEDDS. Notes: (**A**) the P_app_ of CAT3-API, PF403-API, and CAT3-SMEDDS (* *p* < 0.05, ** *p* < 0.01; *n* = 3); (**B**) confocal laser scanning microscopy of Caco-2 monolayers after 2-h incubation with Cou-6 solution or Cou-6-loaded self-microemulsifying drug delivery system (SME) (100 ng Cou-6 equiv./mL; scale bar 10 μm). Abbreviations: CAT3-API, CAT3 suspension; PF403-API, PF403 suspension; CAT3-SMEDDS, CAT3-loaded self-microemulsifying drug delivery system; Cou-6, Coumarin 6; API, Cou-6 suspension; SME, Cou-6-loaded self-microemulsifying drug delivery system; DAPI, 4′,6′-diamidino-2-phenylindole.

The transepithelial transport of CAT3-SMEDDS, CAT3, and PF403 across the MDCK-MDR1 cell monolayer was determined. The results are summarized in Table 1 and Figure 4A. Both CAT3 and PF403 exhibited a very low *P*_app(AP-BL)_ [(0.1056 ± 0.0289) × 10^−6^ and (0.1617 ± 0.0345) × 10^−6^ cm/s, respectively] in the MDCK-MDR1 cell model at 100 ng/mL. However, PF403 exhibited a strong efflux in this cell model. The secretory permeability coefficient of PF403 [*P*_app(BL-AP)_: (0.4877 ± 0.1571) × 10^−6^ cm/s] was found to be 3-fold higher than its absorptive permeability coefficient [*P*_app(AP-BL)_: (0.1617 ± 0.0345) × 10^−6^ cm/s]. CAT3 did not show an observable efflux profile at the same time. It is worth mentioning that *P*_app(AP-BL)_ of CAT3-SMEDDS was 3.9-fold higher than CAT3.

### 2.5. In Vitro Cellular Uptake of SMEDDS

The in vitro cellular uptake profile of Coumarin-6 (Cou-6)-encapsulated SMEDDS was qualitatively analyzed by using confocal laser scanning microscopy. As shown in Figure 4B, the fluorescence intensity of Caco-2 cells incubated with Cou-6-encapsulated SMEDDS was stronger than that of the Cou-6 solution. However, the uptake of Cou-6-encapsulated SMEDDS was less when incubated at 4 °C compared with incubation at 37 °C, indicating that the enhancement of cellular uptake by SMEDDS was energy-dependent.

### 2.6. In Vivo Plasma Pharmacokinetic and Brain Distribution Study

**Figure 5:**
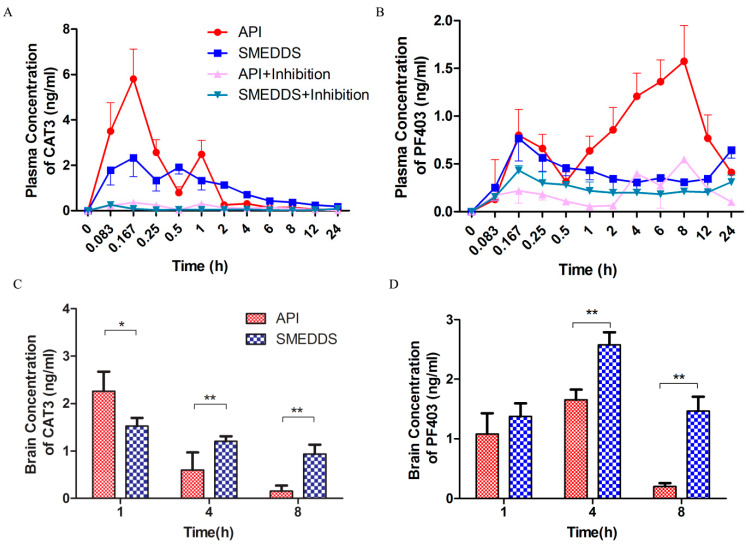
Pharmacokinetic analysis of CAT3-SMEDDS. Notes: (**A**) plasma concentration vs. time profiles of CAT3 (as the pro-drug form) after oral administration; (**B**) plasma concentration vs. time profiles of PF403 (as the metabolite); (**C**) concentration of CAT3 (as the pro-drug form) in the brain after oral administration; (**D**) concentration of PF403 (as the metabolite) in the brain after oral administration (* *p* < 0.05, ** *p* < 0.01; *n* = 3). Abbreviations: API, CAT3 suspension; SMEDDS, CAT3-loaded self-microemulsifying drug delivery system.

The plasma concentration–time curve after the oral administration of the CAT3 API suspension or CAT3-SMEDDS is shown in Figure 5A,B, and the main pharmacokinetic parameters are listed in Table 2 and Table 3. The relative AUC values are shown in Table 4. In Figure 5A, a double absorption peak is observed in the CAT3 suspension, which may be a result of enterohepatic circulation. The AUC_0~t_, C_max_, and MRT_0~t_ values of CAT3 in the group treated with CAT3-SMEDDS were 1.79-, 0.40-, and 1.72-fold higher than those in the group treated with the CAT3 suspension. The AUC_0~t_, C_max,_ and MRT_0~t_ values of PF403 in the group treated with CAT3-SMEDDS were 0.49-, 0.49-, and 1.44-fold higher than those in the group treated with CAT3.

The concentrations of CAT3 and its metabolite, PF403, in the brain tissue at different times are shown in Figure 5. As shown in Figure 5C, the concentration of CAT3 in the group treated with CAT3-SMEDDS was lower (67%) than that in the group treated with the CAT3 API suspension at 1 h. However, it was 2- and 6-fold higher than that of CAT3 at 4 and 8 h, respectively. Furthermore, the concentrations of PF403 in the brain tissue were increased with the administration of CAT3-SMEDDS at all the time points, which were 1.27-, 1.56-, and 7.19-fold higher than those with the administration of CAT3 (Figure 5D). The difference was statistically significant at 4 and 8 h.

As shown in Figure 5A,B, the AUC_0~t_ of CAT3-SMEDDS and CAT3 decreased significantly in chylomicron flow-blocked rats, for both CAT3 and the metabolite PF403, indicating that chylomicron flow is the main absorption pathway for CAT3.

### 2.7. In Vivo Anti-Glioma Effect of CAT3-SMEDDS

**Figure 6:**
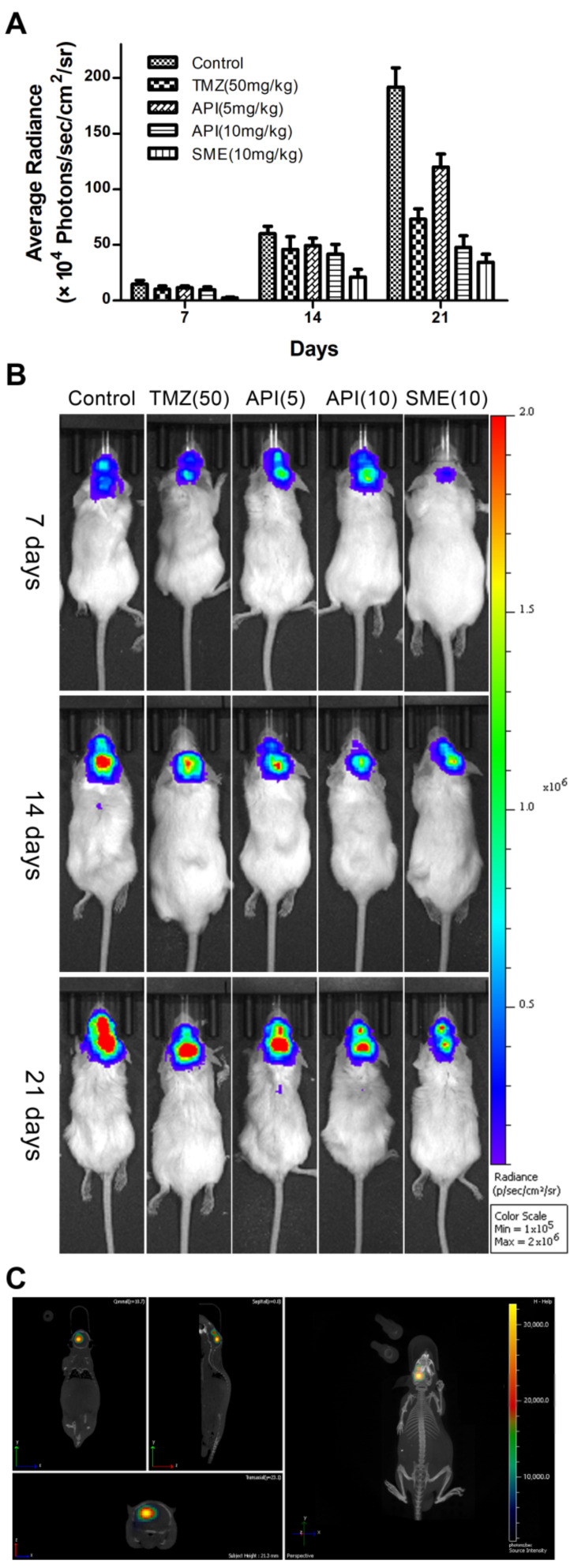
Anti-glioma efficacy of CAT3-SMEDDS in C6-luc orthotopic mice. Notes: (**A**) quantitative image analysis of the intensity of light emitted from intracranial tumor sites (*n* = 5); (**B**) bioluminescent imaging in C6-luc orthotopic mice; (**C**) 3D reconstruction imaging of C6-luc orthotopic mouse in vivo. Abbreviations: TMZ, temozolomide; API, CAT3 suspension; SMEDDS, CAT3-loaded self-microemulsifying drug delivery system.

The in vivo anti-glioma study was performed in an orthotopic mice model. The bioluminescence signals were monitored and quantified at predetermined time points to evaluate the tumor inhibitory effects of SMEDDS, as shown in Figure 6. The results showed that the anti-glioma effect of CAT3-SMEDDS was significantly stronger than that of the CAT3 suspension at the same dose.

### 2.8. Preliminary Safety Evaluation

**Figure 7:**
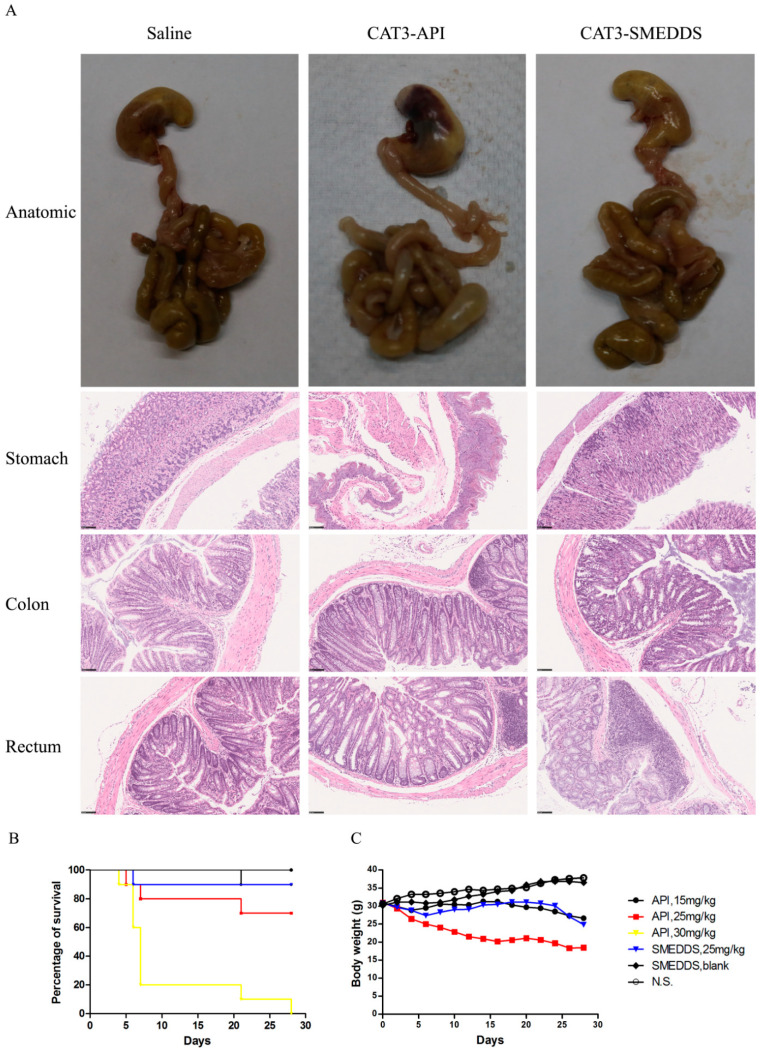
In vivo preliminary toxicity. Notes: the gastrointestinal anatomic images and photomicrograph of hematoxylin and eosin staining of the stomach, colon, and rectum of mice after administration of saline, 25 mg/kg/d CAT3-API, and 25 mg/kg/d CAT3-SMEDDS (**A**); the survival ratio of ICR mice (**B**); the body weight of ICR mice (**C**). Black bar: 100 μm. Abbreviations: API, CAT3 suspension; SMEDDS, CAT3-loaded self-microemulsifying drug delivery system.

As shown in Figure 7B, the orally administered CAT3 suspension exhibited a clear dose–survival rate relationship in mice. At 30 mg/kg/day CAT3 suspension, there was only about an 80% death rate on day 7. However, the death rate at 25 mg/kg/day CAT3-SMEDDS was lower than that at the same dose of CAT3, which was roughly equivalent to 15 mg/kg/day CAT3. The stomach and the intestine images of the mice at day 15 after the first administration of saline, 25 mg/kg/d CAT3-API suspension, or 25 mg/kg/d CAT3-SMEDDS are shown in Figure 7A. There are black coloration and necrosis in the stomachs and obvious edema in the intestines of mice in the CAT3 group. However, the stomachs of the group treated with CAT3-SMEDDS are normal, and only light edema can be observed in the intestine. The stomach and the intestine of the saline and blank SMEDDS groups are normal. The toxicity of CAT3 to the stomach and rectum of the mice can be more clearly displayed in pathological sections. As shown in Figure 7A, H&E staining results show that inflammatory cell infiltration and bleeding spots were seen in the animal stomach after administration of CAT3 for 14 days, indicating that it had a stimulating effect on the stomach. It can be seen from the colon pathological results that obvious infiltration of inflammatory cells occurred which was accompanied by a significant increase in the connective tissue gap in the CAT3 group. More importantly, the infiltration of inflammatory cells in the rectal area of animals in the CAT3 group was more severe. However, the status of the stomach, colon, and rectum in the CAT3-SMEDDS group was better than that of the CAT3 group.

Meanwhile, the body weight–time curve also demonstrates that the weights of the mice that received CAT3-SMEDDS were less affected than those of mice that received the CAT3 suspension (Figure 7C). Moreover, loose stool and diarrhea were found at days 3–28 after the administration of the CAT3 suspension, but there was only soft stool from the third day after the administration of CAT3-SMEDDS, and diarrhea was not found during the whole study period. The stools of the rats in the saline and blank SMEDDS groups were normal. Taken together, this study demonstrates that CAT3-SMEDDS can reduce the gastrointestinal side effects of CAT3.

## 3. Discussion

There are established mature SMEDDS products on the market [25], such as cyclosporine A SMEDDS Neoral^®^ (Novartis, Basel, Switzerland) [26]. The CAT3-SMEDDS developed in this study disperses easily in water, and the droplet size is about 30 nm. Due to the tertiary nitrogen in the molecular structure of CAT3, there is a weak positive charge on the emulsion droplets in water.

CAT3 is not stable in gastrointestinal fluid. It was reported that 59% CAT3 was metabolized into PF403 within 15 min, after incubation with artificial intestinal fluid [8]. When CAT3 was encapsulated in SMEDDS emulsion droplets, it was separated from the digestive juice. Up to 91% and 97% of CAT3 were left after incubation with artificial gastric and intestinal fluid for 4 h, respectively.

The P_app(AP-BL)_ of CAT3 was increased about 3.91 times after being encapsulated in CAT3-SMEDDS. Furthermore, the efflux ratio (ER), which represents the ratio of P_app(BL-AP)_ vs. P_app(AP-BL)_, was reduced. This is an important factor that promotes the oral absorption of CAT3, as a BCS IV compound. Being nanosized, having a positive charge, and having a huge surface facilitate the absorption of the droplets by negatively charged mucosa [27,28]. Furthermore, the ER of PF403 was significantly higher than that of CAT3, indicating that PF403 is a P-gp substrate. Since SMEDDS reduced the generation of PF403 in the gastrointestinal tract and plasma, the efflux amount of the active ingredient also decreased, causing an improvement in the bioavailability of CAT3.

SMEDDS, as a lipid-based formulation, is easily absorbed and transported through the small intestine lymphatic transport. According to the results of the intestinal lymphatic transport of CAT3-SMEDDS in rats which was assessed using a chylomicron flow blocking approach [29], they demonstrate that CAT3-SMEDDS is mainly absorbed (about 88%) through the small intestine lymphatic transport. This route decreases the liver first-pass effect and plays an important role in the plasma metabolism modification effect. Therefore, CAT3-SMEDDS increased the AUC of CAT3 in plasma compared to the CAT3 suspension. In addition, the intestinal lymph flow was very slow [30], the Cmax decreased, and MRT was prolonged, demonstrating a controlled release effect. The sustained release profile of CAT3 can facilitate the entrance of more active substances into the brain tissue, prolong the residence time, and increase the anti-glioma effect. Moreover, the avoidance of the liver first-pass effect decreases the chance of CAT3 degradation by lipase and reduces the exposure amount of PF403 in plasma.

Compared with the CAT3 suspension, SMEDDS significantly promoted the anti-tumor effect in orthotopic glioblastoma mice models, which is associated with the increased concentration and retention time of CAT3 and PF403 in the brain.

Compared with the CAT3 suspension, the encapsulation effect of SMEDDS also separates CAT3 from the gastrointestinal mucosa, together with the reduction in PF403 in the gastrointestinal tract and plasma, which helps to alleviate gastrointestinal side effects.

Finally, compared with OA-CAT3-SLN, CAT3-SMEDDS shows the following advantages. The controlled release of CAT3 from CAT3-SMEDDS in vitro is the slowest compared with the CAT3 suspension and OA-CAT3-SLN [9]. Thus, this controlled release will make the encapsulated CAT3 more stable not only in the gastrointestinal fluid but also in plasma. Next, the results of the transepithelial transport study show that there are no obvious differences between the P_app(AP-BL)_ of CAT3-SMEDDS (0.4129 ± 0.1103 × 10^−6^ cm/s) and OA-CAT3-SLN (0.2557 ± 0.0578 × 10^−6^ cm/s) [9], which means that both of these two DDSs can promote the transepithelial transport. Furthermore, the metabolite style in the plasma between CAT3-SMEDDS and OA-CAT3-SLN was different. It was reported that the bioavailability of CAT3 (as the pro-drug in plasma) was decreased while PF403 (as the metabolite in plasma) was increased after administration of OA-CAT3-SLN. This may be due to the fast CAT3 release from OA-CAT3-SLN, following the fast absorption into the blood circulation system and transformation to PF403 by lipase in plasma. Different to OA-CAT3-SLN, CAT3 was well encapsulated by SMEDDS and was absorbed through the small intestine lymphatic transport, following the completely opposite metabolic phenomenon.

## 4. Materials and Methods

### 4.1. Materials

CAT3 (purity > 99%), PF403 (purity > 99%), and CAT (purity > 99%) were prepared in our institute (Institute of Materia Medica, Chinese Academy of Medical Sciences & Peking Union Medical College, Beijing, China), and the structures were confirmed by infrared, two-dimensional nuclear magnetic resonance and Fourier transform mass spectrometry. Temozolomide and cycloheximide were purchased from J&K Chemical (Beijing, China). Isopropyl myristate (IPM) and macrogolglycerol ricinoleate (Chremfol EL) were gifts from BASF (Ludwigshafen, Germany). Octyl/decyl mono- and diglycerides (Labrasol) were gifts from Gattefosse (Saint-Priest, CEDEX, France). Solvents for high-performance liquid chromatography (HPLC) were obtained from Thermo Fisher Scientific (Waltham, MA, USA). All other chemicals used were analytical grade.

### 4.2. Cell Culture

MDCK-MDR1 cells expressing a high level of P-gp were generously provided by Professor Li (Institute of material medica, PUMC, China). Luciferase-expressing C6 cells (C6-luc) were generously provided by Professor Huang (Institute of material medica, PUMC, People’s Republic of China). Caco-2 cells were purchased from the Cell Resource Center, Peking Union Medical College (Beijing, China). MDCK-MDR1, C6, and Caco-2 cells were cultured in Dulbecco’s Modified Eagle’s Medium (Hyclone, Logan, UT, USA) supplemented with 10% fetal bovine serum (Gibco, ThermoFisher Scientific) and 1% penicillin/streptomycin (Gibco, ThermoFisher Scientific) under 5% CO_2_ at 37 °C.

### 4.3. Animals

ICR mice (20–25 g) and Sprague Dawley rats (males, 200–250 g) were purchased from Beijing Vital River Laboratory Animal Technology (Beijing, China) and were raised at the Institute of Material Medica, Chinese Academy of Medical Sciences and Peking Union Medical College (Beijing, China). All animal experiments were performed in accordance with the guidelines of laboratory animals—guidelines for ethical review of animal welfare (GB_T 35892.2018), for the welfare of the animals.

### 4.4. Preparation and Characterization of CAT3-SMEDDS

CAT3-SMEDDS was composed of IPM (30%, *w*/*w*), Cremophor EL (52.5%, *w*/*w*), and Labrasol (17.5%, *w*/*w*). Preparation of CAT3-SMEDDS was consisted of simply mixing the components, and the mixture was sealed in a glass vial and stored under room temperature. The formulation and preparation processes of CAT3-SMEDDS were optimized based on self-emulsifying efficiency, average droplet size, PDI, and zeta potential.

The dispersibility of CAT3-SMEDDS was tested by diluting the formulation 10–1000 times with distilled water at 37 °C, with constant magnetic stirring at 50 rpm. CAT3-SMEDDS was observed for the formation of stable nanoemulsions. Next, they were visually observed for phase clarity, self-emulsification time, and rate of emulsification.

Average droplet size, PDI, and zeta potential of CAT3-SMEDDS were determined by a dynamic laser light scattering analyzer (Malvern Zetasizer^®^ Nano ZS 90; Malvern Instruments, Malvern, UK).

In order to determine the effects of different dilution volumes on the droplet size of microemulsions, 1 g of CAT3-SMEDDS was added into 10, 20, 50, 100, 500, and 1000 mL of distilled water, and the resulting solutions were slightly shaken. The droplet sizes of these solutions were analyzed.

The effect of different drug loadings on droplet size and media was studied; 0, 1, 3, 5, or 10 mg of CAT3-API powder was added to 1 g of blank SMEDDS to prepare different concentrations of CAT3-SMEDDS. The solutions of CAT3-SMEDDS were diluted in 100 mL distilled water or SGF, followed by the determination of droplet size and zeta potential. All analyses were performed in triplicate.

The morphological evaluation of CAT3-SMEDDS was performed using TEM (JEM-1400 plus; JEOL, Tokyo, Japan). CAT3-SMEDDS was diluted with distilled water at 1:100 and mixed by slight shaking. One drop of the diluted samples was deposited on a film-coated copper grid and allowed to dry before observation under the electron microscope.

### 4.5. Stability of CAT3-SMEDDS in Artificial Gastrointestinal Fluid

The stability of CAT3-SMEDDS in artificial gastrointestinal fluids was performed by incubating CAT3-SMEDDS in SGF and SIF, individually, under constant stirring with a magnetic stirrer at 100 rpm. SGF was prepared according to USP specifications (Test Solutions, United States Pharmacopeia 35, NF 30, 2012). Sodium chloride was added to a 200 mL flask and dissolved in 100 mL of water. Next, 1.4 mL of 10 M HCl was added to adjust the pH of the solution to 1.2. To this, 0.64 g of pepsin was added and dissolved with gentle shaking and the volume was adjusted to up to 200 mL with distilled water. Pepsin was added into the system only after the pH was adjusted to 1.2. SIF was also prepared according to USP specifications (Test Solutions, United States Pharmacopeia 35, NF 30, 2012). Monobasic potassium phosphate was dissolved in 50 mL of water, and 15.4 mL of 0.2 M NaOH was added to adjust the solvent’s pH to 6.8. To this, 2 g of pancreatin was added and shaken gently until dissolved and the volume was adjusted to 200 mL with distilled water. To avoid precipitation of the enzyme, pancreatin was added after adjusting the pH of the solution to 6.8. Next, 0.1 g of CAT3-SMEDDS (loaded with 0.1 mg CAT3) was added into 25 mL SGF or SIF in 50 mL flask, individually, followed by incubation at 37 °C and stirring at 100 rpm for 8 h. All incubations were performed in triplicate. Samples (1 mL) were withdrawn at 0, 1, 2, 4, and 8 h. All the samples were filtered with a 0.22 μm filter membrane and analyzed quantitatively by HPLC.

### 4.6. In Vitro Release

Drug release from CAT3-SMEDDS was monitored in vitro under simulated gastrointestinal conditions. The release behavior of CAT3 was compared under the same condition. Tests were carried out by placing 1 g CAT3-SMEDDS with different concentrations of CAT3 (1 mg and 10 mg/mL) into 900 mL dissolution medium maintained at 37 °C. Release behaviors were studied by using a dissolution instrument (ZRD-8B dissolution tester, Tianda Tianfa Co., Tianjin, China). The dissolution media contained 0.5% sodium dodecyl sulfate (SDS) (*w*/*v*) to meet sink conditions. SDS solution (5 mL, 0.5% *w*/*v*) in preswollen 8–10 kDa MWCO dialysis bags (Sigma-Aldrich, St. Louis, MO, USA) was placed into the same dissolution flask at the same time (*n* = 3). The samples (1 mL) were withdrawn at predetermined times from the media and replaced with the same amount of fresh medium maintained at 37 °C. The samples were centrifuged for 5 min at 12,000 rpm, and the resultant supernatants were quantified for drug content. Samples were protected from light during the experiments. DDsolver software was used to evaluate drug release kinetics [31,32].

### 4.7. Bidirectional Transport of CAT3-SMEDDS in MDCK-MDR1 Monolayer

The permeabilities of CAT3, PF403, and CAT3-SMEDDS were also investigated on a MDCK-MDR1 cell monolayer. MDCK-MDR1 cells were seeded onto each 12-well Transwell^®^ filter insert (pore size 0.4 μm, surface area 1.12 cm^2^; Corning Incorporated, Corning, NY, USA) at a density of 2 × 10^5^ cells/well. The culture medium was changed every 24 h for 7 days, and the cell monolayers with a transepithelial electrical resistance (TEER) above 110 Ω·cm^2^ were used for the transport experiments. The culture medium was removed, and the monolayer was pre-incubated with 0.5 mL of Hanks’ balanced salt solution (HBSS) for 20 min at 37 °C.

After measuring TEER, HBSS was removed, and 0.5 mL of 100 ng/mL CAT3, PF403, or CAT3-SMEDDS diluted with HBSS was added to the apical side (AP), and 1.5 mL of HBSS was added to the basolateral (BL) compartment. Samples (50 μL) were taken from the BL compartment at 0.5, 1, 1.5, and 2 h. The samples were stored at 4 °C for analysis. The *P*_app_ from BL to AP was determined by adding 1.5 mL samples to the BL side and 0.5 mL of HBSS to the AP side.

The concentration of CAT3, PF403, or CAT3-SMEDDS permeated through the monolayer was determined using the liquid chromatography-mass spectrometry (LC-MS/MS) system. The apparent permeability coefficient (*P*_app_) of CAT3, PF403, or CAT3-SMEDDS was calculated according to the following equation: *P*_app_ = d*Q*/d*t* × 1/(*A* × *C*_0_), where d*Q*/d*t* indicates the linear appearance rate of mass at the BL side (μmol/s), *C*_0_ is the initial concentration of CAT3, PF403, or CAT3-SMEDDS on the AP side (μmol/mL), and *A* is the surface area of the monolayer (cm^2^).

### 4.8. In Vitro Cellular Uptake of SMEDDS

To verify whether SMEDDS can enhance intracellular delivery, a fluorescence microscope study was conducted. Fluorescent Cou-6 was loaded in SMEDDS [33,34]. Caco-2 cells were seeded in 6-well plates containing cover glass at 5 × 10^4^ cells/well in cell culture medium overnight. Cou-6-loaded SMEDDS or Cou-6 solution in 2 mL of phosphate-buffered saline (PBS; Cou-6 concentration: 100 ng/mL) was added and incubated at 37 °C for 1 h. At the same time, another Cou-6-SMEDDS was incubated at 4 °C for 1 h. After the incubation, the cells were washed three times with cold PBS and fixed with 4% paraformaldehyde for 15 min. The cells were washed three times with cold PBS and stained with 4′,6-diamidino-2-phenylindole. Next, the cells were visualized using a confocal microscope (FV1000; Olympus, Tokyo, Japan).

### 4.9. In Vivo Pharmacokinetic Study in Plasma and Drug Distribution in Brain

For plasma pharmacokinetic studies, 24 male rats were separated into four groups (n = 6 per group). Before the day of administration, the rats were fasted for 12 h but allowed water ad libitum. CAT3 suspension (10 mg/kg dispersed in 0.5% CMC-Na solution) and the same dose of CAT3-SMEDDS were administered orally. One hour before the experiment, two of the groups were treated with intraperitoneal injection of 3 mg/kg cycloheximide solution in saline [29,35] before the administration of 10 mg/kg of CAT3 and CAT3-SMEDDS. Blood samples (150 μL) were collected into heparinized tubes from each rat through the puncture of the retro-orbital sinus. This was performed at 0, 5, 10, 15, and 30 min and 1, 2, 4, 6, 8, 12, and 24 h after oral administration. The blood samples were immediately processed to obtain the plasma by centrifuging at 4000 rpm for 10 min. The plasma was collected and frozen at −80 °C until analysis by LC-MS/MS.

For the brain pharmacokinetic study, 18 rats were randomly divided into two groups for three time points of the study (*n* = 9; 3 animals per time point), and they were administered CAT3 or SMEDDS at a dose of 10 mg/kg via oral administration. Three rats of each group at predetermined time points (1, 4, and 8 h) were sacrificed and the brain tissues were collected, washed, and frozen at −80 °C for LC-MS/MS analysis.

### 4.10. Bioanalysis of CAT3 and PF403 in Rat Plasma and Brain Tissue Using LC-MS/MS

For in vivo analysis of CAT3 and its metabolite, FP403, in rat plasma, 10 μL of the internal standard (ISTD, CAT, molecular structure is shown in Figure 1C, 10 ng/mL) was added to 50 μL of plasma. After the addition of 90 μL of acetonitrile for the precipitation of protein, the sample was vortex-mixed for 30 s, followed by centrifugation at 13,000 rpm for 10 min. Brain samples were homogenized with saline (1:3). An aliquot (50 μL) of the homogenates was precipitated by adding 90 μL of acetonitrile and 10 μL ISTD followed by vortexing for 30 s and centrifugation for 10 min at 13,000 rpm. The supernatant was transferred to a new tube, and a 5-μL aliquot of the solution was injected into the HPLC-tandem mass spectrometry system for analysis.

Triple-quadrupole mass spectroscopy (6410B, Agilent, Santa Clara, CA, USA) under multiple reaction monitoring and positive electrospray ionization mode. Nitrogen (350 °C) as the nebulizer gas was flowed at 10 L/min and the pressure was set to 40 psi. The voltages of the fragmentor potential and collision energy were 135 and 20 eV, respectively. The ion reactions for the determination of CAT3, PF403, and ISTD (CAT) were *m*/*z* 434.3 → *m*/*z* 70.1, 350.2 → 70.1, and 364.2 → 70.1, respectively.

The concentrations of analytes were determined using MassHunter software (Agilent, Santa Clara, CA, USA). Chromatographic separation was performed on a 1200 serious RRLC system (Agilent, USA) with a C18 column (50 × 2.1 mm, 1.8 μm, Agilent Santa Clara, CA, USA) and a corresponding guard column (ODS, 5 μm). Distilled water and 0.1% formic acid acetonitrile (20:80, *v*/*v*) were used as the mobile phase for elution. The flow rate was 0.4 mL/min at 40 °C.

The primary pharmacokinetic parameters were calculated using Drug and Statistics (DAS) 3.0 software (BioGuider Medicinal Technology Co. Ltd., Shanghai, China). The non-compartmental model was used to estimate the pharmacokinetic parameters.

### 4.11. In Vivo Anti-Glioma Effect of CAT3-SMEDDS

The in vivo anti-glioma efficacy was evaluated on male ICR mice bearing C6-luc orthotopic glioblastoma. All surgical instruments and supplies were sterilized before use. The mice were anesthetized with 40 mg/kg pentobarbital sodium, immobilized in a small animal stereotaxic apparatus (Beijing Zhishuduobao Biological technology Co. LTD., Beijing, China), and placed on a heating pad at 37 ± 1 °C to maintain their body temperature. A skin incision was made on the top of the skull after antisepsis and a burr hole was drilled into the skull of the right hemisphere. Next, 2 × 10^5^ C6-luc suspended in 4 μL of PBS was injected into the right striatum through the hole using a 10 μL needle driven by a micro syringe pump (ALC-IP, Shanghai Alcott Biotech Co. LTD., Shanghai, China) at a speed of 0.8 μL/min. After seeding, the needle was retained for 2 min and then drawn out carefully and slowly. Finally, paraffin was used to fill the surgical hole and the incision was sutured. The mice were closely observed until they were completely awake [36].

The bioluminescent imaging was performed with IVIS Spectrum CT (Caliper-Perkin Elmer, Waltham, MA, USA). On the 4th day after surgery, the mice were administered d-Luciferin substrate in PBS (150 mg/kg) via intraperitoneal injection. At 15 min post-injection, the mice were anesthetized with isoflurane and subsequently imaged with the IVIS system. Average luminescence intensity (photon/s) was quantified by analyzing the head region with a software package. Twenty-five mice with C6-luc orthotopic models were randomly divided into five groups (*n* = 5 per group) by the luminescence intensity. Subsequently, the mice were administered normal saline (control group), TMZ (50 mg/kg/day), CAT3 (5 mg/kg/day), CAT3 (10 mg/kg/day), and CAT3-SMEDDS (10 mg/kg/day) orally for 21 days. At the end of the 7th, 14th, and 21st days after oral administration, the luminescence intensity was measured for all the groups.

### 4.12. Preliminary In Vivo Safety Evaluation

To assess the safety and the side effects of the different doses of CAT3-SMEDDS, CAT3, and blank SMEDDS, the body weight, survival curve, and stool status of the mice were recorded. Sixty mice were randomly divided into six groups (*n* = 10 per group). Subsequently, the mice were administered saline (control group), CAT3 (15, 25, and 30 mg/kg/day), CAT3-SMEDDS (25 mg/kg/day), and blank SMEDDS (the same dose as CAT3-SMEDDS) orally for 28 days. The body weights of all the mice were recorded every two days over the entire treatment period. The survival status and the stool status were observed every day. For the histological evaluation study, 12 mice were randomly divided into 4 groups (*n* = 3 per group) and orally given saline (control group), CAT3 (15, 25, and 30 mg/kg/day), CAT3-SMEDDS (25 mg/kg/day), and blank SMEDDS, individually, for 14 days. At the end of the study, all mice were euthanized by cervical dislocation and the entire stomach and intestines of the male mice were immediately removed and checked by visual observation. Next, the stomach, colon, and rectum organs were flushed with saline gently, cleaned of fat and mesentery on ice-cold plates, and patted dry using filter paper. After that, the washed stomach, colon, and rectum samples were fixed for 48 h in 4% paraformaldehyde, washed in pH 6.4 phosphate buffer, and then embedded in paraffin blocks. The 4 μm slices were sectioned and stained by hematoxylin and eosin (H&E) for histological evaluation.

### 4.13. Statistical Analyses

All data are expressed as mean ± standard deviation. A *p*-value less than 0.05 indicates statistical significance using a *t*-test between two mean values for unpaired data.

## 5. Conclusions

SMEDDS is a suitable drug delivery system for the encapsulation and delivery of CAT3. The encapsulation effect of SMEDDS not only separates CAT3 from the gastrointestinal mucosa but also reduces the generation of its metabolite, PF403, in the digestive tract and plasma, significantly reducing the gastrointestinal side effects.

In addition, SMEDDS showed a beneficial metabolism modification effect on the pharmacokinetic properties of CAT3 in the plasma and brain. It increased the bioavailability of CAT3 and reduced the exposure amount of its metabolite, PF403, in the plasma. Furthermore, the concentration and residence time of both CAT3 and PF403 in the brain tissue were significantly increased. The metabolism modification function of SMEDDS improved the anti-tumor effect of CAT3 in an orthotopic glioma model.

## Figures and Tables

**Table 1:** The apparent permeability coefficient (Papp) of CAT3, PF403, and CAT3-SMEDDS.

Name	Papp (×10^−6^ cm/s)				ER
	AP-BL	%	BL-AP	%	
CAT3	0.1056 ± 0.0289	100	0.1021 ± 0.0154	100.0	0.9662
PF403	0.1617 ± 0.0345	153.2	0.4877 ± 0.1571	477.7	3.0155
CAT3-SMEDDS	0.4129 ± 0.1103	391.0	0.2411 ± 0.1239	236.2	0.5840

**Table 2:** The pharmacokinetic parameters of CAT3 (as the pro-drug form) in rats after oral administration of CAT3 or SMEDDS.

Parameter		CAT3-API	CAT3-SMEDDS	CAT3-API+ Inhibition	SMEDDS+ Inhibition
AUC_(0–t)_	ng/(mL·h)	5.911 ± 0.396	10.564 ± 0.154	1.862 ± 0.356	1.235 ± 0.166
AUC_(0–∞)_	ng/(mL·h)	7.369 ± 0.625	12.499 ± 1.928	1.971 ± 0.242	1.616 ± 0.645
MRT_(0–t)_	H	4.208 ± 0.456	7.247 ± 0.08	7.369 ± 0.669	14.498 ± 0.429
MRT_(0–∞)_	H	13.033 ± 6.792	12.18 ± 4.651	9.042 ± 1.923	24.766 ± 17.932
t_1/2z_	H	15.472 ± 6.988	10.013 ± 4.243	5.364 ± 2.704	37.115±
Tmax	H	0.167 ± 0	0.167 ± 0	0.167 ± 0	0.083 ± 0
Vz/F	L/kg	29,688.6957 ± 11,885.0772	11,261.752 ± 2910.449	41,374.9424 ± 26,075.1155	22916.9716±
CLz/F	L/h/kg	1363.91521 ± 121.36206	811.881 ± 115.054	5128.74435 ± 674.216985	6823.5499 ± 2437.732
Cmax	ng/mL	5.811 ± 0.313	2.331 ± 0.022	0.361 ± 0.012	0.257 ± 0.043

**Table 3:** The pharmacokinetic parameters of PF403 (as the metabolite) in rats after oral administration of CAT3 or SMEDDS.

Parameter		CAT3-API	CAT3-SMEDDS	CAT3-API+ Inhibition	SMEDDS+ Inhibition
AUC_(0–t)_	ng/(L·h)	20.537 ± 0.256	10.008 ± 0.677	5.747 ± 0.333	5.542 ± 0.455
AUC_(0–∞)_	ng/(L·h)	26.578 ± 0.658	13.397 ± 6.082	7.018 ± 0.974	84.891 ± 81.228
MRT_(0–t)_	h	9.73 ± 0.106	14.045 ± 0.42	9.998 ± 0.222	13.269 ± 0.828
MRT_(0–∞)_	h	16.373 ± 1.002	22.828 ± 15.377	14.785 ± 2.65	403.858 ± 404.671
t_1/2z_	h	10.348 ± 0.982	29.824 ± 1.996	8.602 ± 2.642	416.244 ± 218.378
Tmax	h	8 ± 0	0.167 ± 0	8 ± 0	0.167 ± 0
Vz/F	L/kg	5611.71866 ± 390.472773	21,117.8511 ± 1760.5231	17,410.146 ± 2991.1324	47,975.427 ± 1349.4173
CLz/F	L/h/kg	376.406394 ± 9.18153	839.472493 ± 309.367699	1443.4282 ± 200.42835	614.21329 ± 905.19275
Cmax	ng/L	1.574 ± 0.075	0.766 ± 0.036	0.548 ± 0.021	0.431 ± 0.05

**Table 4:** The relative AUC_(0–t)_ in rats after oral administration of CAT3 or SMEDDS.

Group	CAT3 in Plasma		Metabolite PF403 in Plasma	
	AUC_(0–t)_ ng/L·h	Relative AUC_(0–t)_(%)	AUC_(0-t)_ ng/L·h	Relative AUC_(0–t)_(%)
CAT3	5.911 ± 0.396	100.00	20.537 ± 0.256	100.00
SMEDDS	10.564 ± 0.154 **	178.72	10.008 ± 0.677 **	48.73
CAT3 + Inhibition	1.862 ± 0.356 **	31.50	5.747 ± 0.333 **	29.98
SMEDDS + Inhibition	1.235 ± 0.166 ^##^	11.69 ^Note 1^	5.542 ± 0.455 ^## Note 1^	55.38 ^Note 1^

** *p* < 0.01 vs. CAT3-API, ^##^ *p* < 0.01 vs. CAT3-SMEDDS. Note 1: AUC _(0–t)_ ng/L·h vs. SMEDDS.

## Data Availability

Data is contained within the article. The data presented in this study are available in Improved Safety and Anti-Glioblastoma Efficacy of CAT3-Encapsulated SMEDDS through Metabolism Modification.

## Sample Availability

The sample of compounds CAT3 and PF403 are available from authors.

## Abbreviations

CAT3-SMEDDS	CAT3-loaded self-microemulsifying drug delivery system
GBM	glioblastoma multiforme
CNS	central nervous system
TMZ	temozolomide
SMEDDS	self-microemulsifying drug delivery system
API	active pharmaceutical ingredient
HPLC	high-performance liquid chromatography
C6-luc	luciferase-expressing C6 cells
DI	deionized
PDI	polydispersity index
SGF	simulated gastric fluid
SIF	simulated intestinal fluid
HBSS	Hanks’ balanced salt solution
AP	apical side
BL	basolateral
LC-MS/MS	liquid chromatography-mass spectrometry
Papp	apparent permeability coefficient
PBS	phosphate-buffered saline
Cou-6	fluorescent Coumarin 6
ISTD	internal standard
DAS	Drug and Statistics
ER	efflux ratio

## References

[1] Alifieris C., Trafalis D.T. (2015). Glioblastoma multiforme: Pathogenesis and treatment. Pharmacol. Ther..

[2] Nabors L.B., Portnow J., Ammirati M., Baehring J., Brem H., Brown P., Butowski N., Chamberlain M.C., Fenstermaker R.A., Friedman A. (2015). Central Nervous System Cancers, Version 1.2015. J. Natl. Compr. Cancer Netw..

[3] Ji M., Wang L., Chen J., Xue N., Wang C., Lai F., Wang R., Yu S., Jin J., Chen X. (2018). CAT3, a prodrug of 13a(S)-3-hydroxyl-6,7-dimethoxyphenanthro[9,10-b]-indolizidine, circumvents temozolomide-resistant glioblastoma via the Hedgehog signaling pathway, independently of O6-methylguanine DNA methyltransferase expression. OncoTargets Ther..

[4] Liu Z., Lv H., Li H., Zhang Y., Zhang H., Su F., Xu S., Li Y., Si Y., Yu S. (2011). Interaction Studies of an Anticancer Alkaloid, (+)-(13aS)-Deoxytylophorinine, with Calf Thymus DNA and Four Repeated Double-Helical DNAs. Chemotherapy.

[5] Lv H., Ren J., Ma S., Xu S., Qu J., Liu Z., Zhou Q., Chen X., Yu S. (2012). Synthesis, Biological Evaluation and Mechanism Studies of Deoxytylophorinine and Its Derivatives as Potential Anticancer Agents. PLoS ONE.

[6] Tian Y., He J., Zhang R., Lv H., Ma S., Chen Y., Yu S., Chen X., Wu Y., He W. (2012). Integrated rapid resolution liquid chromatography–tandem mass spectrometric approach for screening and identification of metabolites of the potential anticancer agent 3,6,7-trimethoxyphenanthroindolizidine in rat urine. Anal. Chim. Acta.

[7] Yu S., Yu P., Lv H., Li C., Ren J., Ma S., Xu S., Chen X. (2012). Stereospecific Synthesis and Biological Evaluation of Monodesmethyl Metabolites of (+)-13a-(S)-Deoxytylophorinine as Potential Antitumor Agents. Synthesis.

[8] Li C., Li Y., Lv H., Li S., Tang K., Zhou W., Yu S., Chen X. (2015). The novel anti-neuroblastoma agent PF403, inhibits proliferation and invasion in vitro and in brain xenografts. Int. J. Oncol..

[9] Wang H., Li L., Ye J., Wang R., Wang R., Hu J., Wang Y., Dong W., Xia X.-J., Yang Y. (2020). Improving the Oral Bioavailability of an Anti-Glioma Prodrug CAT3 Using Novel Solid Lipid Nanoparticles Containing Oleic Acid-CAT3 Conjugates. Pharmaceutics.

[10] Cabeza L., Ortiz R., Prados J., Delgado A.V., Martín-Villena M.J., Clares B., Perazzoli G., Entrena J.M., Melguizo C., Arias J.L. (2017). Improved antitumor activity and reduced toxicity of doxorubicin encapsulated in poly(ε-caprolactone) nanoparticles in lung and breast cancer treatment: An in vitro and in vivo study. Eur. J. Pharm. Sci..

[11] Liang Q., Zhang L., Wang T., Li Q., Huang J., Xu H., Li J., Wang Y. (2016). Fabrication of novel vesicles of triptolide for antirheumatoid activity with reduced toxicity in vitro and in vivo. Int. J. Nanomed..

[12] Osman A.-M.M., Al-Kreathy H.M., Al-Zahrani A., Ahmed O., Ramadan W.S., ElShal M.F., Al-Harthi S.E., Ali A.S., Khan L.M. (2017). Enhancement of Efficacy and Reduced Toxicity of Cisplatin Through Self Nanoemulsifying Drug Delivery System (SNEDDS). Int. J. Pharmacol..

[13] Kanoujia K., Dewangan C., Masih A., Sinha D., Oraon D., Jaiswal M., Sahu M., Kumari R., Pradhan S., Suman R. (2018). Self Microemulsifying Drug Delivery System (SMEDDS): A Novel Approach to Improve the Therapeutic Efficacy of Orally Administered Drug. Res. J. Pharm. Dos. Forms Technol..

[14] Patel M.H., Mundada V.P., Sawant K.K. (2019). Novel Drug Delivery Approach via Self-Microemulsifying Drug Delivery System for Enhancing Oral Bioavailability of Asenapine Maleate: Optimization, Characterization, Cell Uptake, and In Vivo Pharmacokinetic Studies. AAPS PharmSciTech.

[15] Jaisamut P., Wiwattanawongsa K., Graidist P., Sangsen Y., Wiwattanapatapee R. (2017). Enhanced Oral Bioavailability of Curcumin Using a Supersaturatable Self-Microemulsifying System Incorporating a Hydrophilic Polymer; In Vitro and In Vivo Investigations. AAPS PharmSciTech.

[16] Constantinides P.P. (1995). Lipid Microemulsions for Improving Drug Dissolution and Oral Absorption: Physical and Biopharmaceutical Aspects. Pharm. Res..

[17] Abdalla A., Klein S., Mäder K., Mäder K. (2008). A new self-emulsifying drug delivery system (SEDDS) for poorly soluble drugs: Characterization, dissolution, in vitro digestion and incorporation into solid pellets. Eur. J. Pharm. Sci..

[18] Patel M.H., Sawant K.K. (2019). Self microemulsifying drug delivery system of lurasidone hydrochloride for enhanced oral bioavailability by lymphatic targeting: In vitro, Caco-2 cell line and in vivo evaluation. Eur. J. Pharm. Sci..

[19] Khatri P., Shao J. (2018). Impact of digestion on the transport of dextran-loaded self-emulsified nanoemulsion through MDCK epithelial cell monolayer and rat intestines. Int. J. Pharm..

[20] Patel D., Sawant K.K. (2009). Self micro-emulsifying drug delivery system: Formulation development and biopharmaceutical evaluation of lipophilic drugs. Curr. Drug Deliv..

[21] Chandrakar A., Sahu B., Sahu H., Dewangan J., Kumar N., Singh R., Gupta R., Kumar D., Sahu B., Dewangan K. (2017). Review on the formulation considerations needed to produce a stable Self micro Emulsifying Drug Delivery System (SMEDDS). Res. J. Pharm. Technol..

[22] Joyce P., Dening T.J., Meola T.R., Schultz H.B., Holm R., Thomas N., Prestidge C.A. (2019). Solidification to improve the biopharmaceutical performance of SEDDS: Opportunities and challenges. Adv. Drug Deliv. Rev..

[23] Vishwakarma N., Jain A., Sharma R., Mody N., Vyas S., Vyas S.P. (2019). Lipid-Based Nanocarriers for Lymphatic Transportation. AAPS PharmSciTech.

[24] Kang B.K., Lee J.S., Chon S.K., Jeong S.Y., Yuk S.H., Khang G., Lee H.B., Cho S.H. (2004). Development of self-microemulsifying drug delivery systems (SMEDDS) for oral bioavailability enhancement of simvastatin in beagle dogs. Int. J. Pharm..

[25] Krstić M., Medarević Đ., Đuriš J., Ibrić S., Grumzescu A.M. (2018). Self-nanoemulsifying drug delivery systems (SNEDDS) and self-microemulsifying drug delivery systems (SMEDDS) as lipid nanocarriers for improving dissolution rate and bioavailability of poorly soluble drugs. Lipid Nanocarriers for Drug Targeting.

[26] Ritschel W.A. (1996). Microemulsion technology in the reformulation of cyclosporine: The reason behind the pharmacokinetic properties of Neoral. Clin. Transplant..

[27] Dyawanapelly S., Koli U., Dharamdasani V., Jain R., Dandekar P. (2016). Improved mucoadhesion and cell uptake of chitosan and chitosan oligosaccharide surface-modified polymer nanoparticles for mucosal delivery of proteins. Drug Deliv. Transl. Res..

[28] Salatin S., Khosroushahi A.Y. (2017). Overviews on the cellular uptake mechanism of polysaccharide colloidal nanoparticles. J. Cell. Mol. Med..

[29] Lind M.L., Jacobsen J., Holm R., Müllertz A. (2008). Intestinal lymphatic transport of halofantrine in rats assessed using a chylomicron flow blocking approach: The influence of polysorbate 60 and 80. Eur. J. Pharm. Sci..

[30] Carrière F. (2016). Impact of gastrointestinal lipolysis on oral lipid-based formulations and bioavailability of lipophilic drugs. Biochimie.

[31] Zuo J., Gao Y., Bou-Chacra N., Löbenberg R. (2014). Evaluation of the DDSolver Software Applications. BioMed Res. Int..

[32] Zhang Y., Huo M., Zhou J., Zou A., Li W., Yao C., Xie S. (2010). DDSolver: An Add-In Program for Modeling and Comparison of Drug Dissolution Profiles. AAPS J..

[33] Zhou W.-H., Zhou Y., Wu J., Liu Z., Zhao H., Liu J., Ding J. (2013). Aptamer-nanoparticle bioconjugates enhance intracellular delivery of vinorelbine to breast cancer cells. J. Drug Target..

[34] Li M., Shi K., Tang X., Wei J., Cun X., Chen X., Yu Q., Zhang Z., He Q. (2018). pH-sensitive folic acid and dNP2 peptide dual-modified liposome for enhanced targeted chemotherapy of glioma. Eur. J. Pharm. Sci..

[35] Dahan A., Hoffman A. (2005). Evaluation of a chylomicron flow blocking approach to investigate the intestinal lymphatic transport of lipophilic drugs. Eur. J. Pharm. Sci..

[36] Huang F.-Y.J., Lee T.-W., Chang C.-H., Chen L.-C., Hsu W.-H., Chang C.-W., Lo J.-M. (2015). Evaluation of 188Re-labeled PEGylated nanoliposome as a radionuclide therapeutic agent in an orthotopic glioma-bearing rat model. Int. J. Nanomed..

